# MicroRNA‐122 promotes apoptosis of keratinocytes in oral lichen planus through suppressing VDR expression

**DOI:** 10.1111/jcmm.16418

**Published:** 2021-03-03

**Authors:** Xuejun Ge, Hanting Xie, Lu Wang, Ran Li, Fang Zhang, Jing Xu, Bin Zhao, Jie Du

**Affiliations:** ^1^ Shanxi Province Key Laboratory of Oral Diseases Prevention and New Materials Shanxi Medical University School and Hospital of Stomatology Taiyuan China; ^2^ Department of Endodontics Shanxi Medical University School and Hospital of Stomatology Taiyuan China; ^3^ Department of Pathology Shanxi Medical University Taiyuan China; ^4^ Department of Oral Medicine Shanxi Medical University School and Hospital of Stomatology Taiyuan China; ^5^ Institute of Biomedical Research Shanxi Medical University Taiyuan China

**Keywords:** apoptosis, miR‐122, oral lichen planus, vitamin D receptor

## Abstract

MicroRNA‐122 (miR‐122) is known to be up‐regulated by inflammation to exert a variety of biological functions in hepatocellular carcinoma (HCC)‐derived human cell lines. Vitamin D receptor (VDR) is reported to regulate excessive oral keratinocytes apoptosis which compromises oral epithelial barrier in oral lichen planus (OLP). Although many studies have suggested that miR‐122 is capable of regulating cell apoptosis, its effects on the development of OLP and VDR expression are still unclear. Herein, we demonstrate that miR‐122 expression is increased in the epithelial layer of OLP. Mechanically, transcription factor nuclear factor‐κB (NF‐κB) selectively binds with κB element in the promoter of *miR‐122* to accelerate gene transcription. The up‐regulation of miR‐122 induces cell apoptosis in human oral keratinocytes (HOKs) by targeting VDR mRNA. In *VDR* knockout oral keratinocytes, miR‐122 fails to improve caspase 3 activity and cleaved caspase 3 and poly(ADP‐ribose) polymerase (PARP) levels. Moreover, VDR overexpression is able to reverse lipopolysaccharide (LPS)‐ or activated CD4+ T cell–induced miR‐122 up‐regulation and ameliorate miR‐122‐stimulated caspase 3 activity. Collectively, our results suggest that miR‐122 promotes oral keratinocytes apoptosis in OLP through decreasing VDR expression.

## 1 INTRODUCTION

Oral lichen planus (OLP), a common immune‐mediated disease, shows oral ulceration and persistent inflammation in patients those who experience discomfort and remarkable impairments of oral functioning and life quality.^1^ Albeit OLP is regarded as an inflammatory disorder, malignant transformation rate of OLP is approximately 1.4% within 7 years.^2^ Ulceration, gender and location on the tongue are key risky factors responsible for malignant transformation.^2^ There are six subtypes of OLP in clinic, including atrophic, reticular, papular, plaque, erosive and bullous.^3^ The reticular pattern displays with typical striated, lacy hyperkeratosis.^4^ The erythema of the atrophic pattern may be mistaken for erythroplakia.^4^ The plaque pattern, appeared as multiple elevated plaques, may be misinterpreted as leukoplakic lesions.^5^

The diagnosis of OLP is mainly based on characteristics of clinical features and histological examination.^3^, ^5^ Biopsy may be required if the clinical feature manifests dysplasia or to rule out immunobullous mimickers.^3^ Due to the lack of established cure for OLP, the main purpose of management of this disease is focused on ameliorating oral painful symptoms and improving patients’ oral functioning and life quality.^3^

MicroRNA‐122 (miR‐122), a highly abundant microRNA in vertebrates with conserved sequence, has been reported to exert regulatory functions in cholesterol and fatty acid metabolism, apoptosis, hepatitis C virus (HCV) propagation and hepatocellular carcinoma (HCC) growth.^6^, ^7^, ^8^, ^9^, ^10^, ^11^ MiR‐122 is encoded on chromosome 18 and transcribed to a ~5 kb non‐coding pri‐miRNA in the presence of RNA polymerase II. Pri‐miRNA is then spliced into pre‐miRNA with 66 nucleotides by Drosha. Finally, pre‐miR‐122 is moved into cytoplasm and then processed into its mature 23‐nucleotide miR‐122.^12^ MiR‐122 which is mainly expressed in mature hepatocytes is mediated by numerous transcription factors such as CAAT/enhancer‐binding protein (C/EBPα) and hepatocyte nuclear factors (HNF1α, HNF3β, HNF4α).^13^, ^14^, ^15^ Moreover, miR‐122 is considered to be a circadian metabolic regulator as its status is regulated by Rev‐ErbA alpha.^16^ Mice with germline miR‐122 deletion manifest spontaneous progression to fibrosis and steatosis at birth.^17^ In humans, inhibition of miR‐122 decreases serum cholesterol and triglycerides and HCV viremia.^9^, ^18^, ^19^ Although the roles of miR‐122 in the liver have been well explored, little is known about its function in immune‐associated diseases such as OLP.

Vitamin D receptor (VDR) which is well known as a nuclear hormone receptor regulates the biological functions of vitamin D.^20^ Dependent on vitamin D’s interaction, VDR in the cytoplasm is shuttled into nucleus and binds with vitamin D response elements (VDREs) in the promoter regions of target genes.^20^ A large number of experimental or epidemiological investigations have revealed that deficiency of VDR in humans is closely associated with the development of inflammatory diseases.^21^, ^22^, ^23^, ^24^ Importantly, a high prevalence of VDR reduction has been stated in the diseased tissues of OLP.^21^ In addition, *VDR* gene polymorphisms are also reported in the context of OLP.^25^

Both miR‐122 and VDR are associated with cell apoptosis in several cell types.^24^, ^26^, ^27^ Apoptosis is regarded as a characteristic of OLP leading to the excessive loss of oral keratinocytes.^24^, ^28^ The impairment of oral mucosal epithelial layer caused by apoptosis aggravates bacterial invasion and the following inflammatory response.^29^, ^30^ In this study, we explored the relationships among miR‐122, VDR and cell apoptosis in oral keratinocytes of OLP. Our findings reveal that miR‐122 promotes oral keratinocytes apoptosis in oral lichen planus by targeting VDR.

## 2 METHODS AND MATERIALS

### 2.1 Human samples collection

Human oral biopsies were collected from participants recruited at the Stomatological Hospital affiliated with Shanxi Medical University as described before.^24^ Healthy control tissues were obtained from individuals undergone wisdom teeth extraction. The investigation was approved by the Institutional Ethical Committee of Shanxi Medical University. Written informed consents from volunteers were got as well. More details concerning OLP patients were provided in previous study.^24^

### 2.2 Cell culture

Human oral keratinocytes (HOKs) were from ScienCell Company and cultured in oral keratinocyte medium (OKM). HOKs were challenged with 100 ng/mL Lipopolysaccharide from Porphyromonas gingivalis (LPS‐PG, MilliporeSigma) or the culture medium of activated cluster of differentiation (CD)4+ T cells for 8 hours. CD4+ T cells supernatant accounts for 30% final volumetric concentration. In another experiment, a variety of plasmids or miRNA mimics were transfected into HOKs for 36 hours.

### 2.3 Animal studies

Six‐week‐old wild‐type and VDR^−/−^ C57BL/6 mice were used in this investigation. VDR^−/−^ mice were generated by a strategy reported previously.^31^ This animal protocol was approved by the Institutional Ethical Committee of Shanxi Medical University.

### 2.4 Oral mucosal epitheliums isolation and culture

The whole buccal biopsies isolated from human or mice were treated with 0.25% dispase II for 12 hours. Epithelium and lamina propria layers were separated directly by muscle forceps. Cell culture of primary mouse oral keratinocytes was performed as described previously.^32^ In brief, the oral epithelial layer from mouse buccal tissue was cut into small pieces followed by digestion with trypsin from Thermo Fisher Scientific. The single keratinocytes were cultured with OKM.

### 2.5 CD4+ T cells isolation and stimulation

Peripheral blood samples collected from patients were subjected to Ficoll‐Hypaque density gradient centrifugation. Anti‐CD4 magnetic particles from BD Biosciences were used to isolate CD4+ T cells. Anti‐CD3 and anti‐CD28 antibodies (BD Biosciences) were applied to activate CD4+ T cells as described before.^24^

### 2.6 Plasmids and transfection

IκB Kinase β (IKKβ) plasmids were given by Yanchun Li (University of Chicago). Fragments from the 3′ untranslated region (UTR) of VDR mRNA or hsa‐*miR‐122* gene promoter were amplified by polymerase chain reaction (PCR) and cloned into the pGL3‐Promoter vector. The transfection assays were performed by using Lipofectamin 3000 from Thermo Fisher Scientific. PCR primers sequences were listed in Table S1.

### 2.7 Western blot

Oral keratinocytes were washed three times with cold phosphate‐buffered saline (PBS) and lysed into lysis buffer. After heating, lysates were centrifuged and the supernatants were separated and transferred to PVDF membranes. 5% non‐fat milk was used to block PVDF membranes. Primary and secondary antibodies were used to incubated membranes. Bands were detected with enhanced chemiluminescence (ECL) kit (Thermo Fisher Scientific) and visualized by x‐ray films. Details of primary antibodies were provided in Table S2.

### 2.8 Reverse transcript (RT)‐PCR

Total RNAs or miRNAs from cells and tissues were extracted by Trizol reagent (Thermo Fisher Scientific) or miRNA isolation Kit (QIAGEN), respectively. Prime Script RT Reagent Kits (TaKaRa) or miRNA First‐strand cDNA Synthesis Kits (Aidlab Biotechnologies) were used to synthesize the first‐strand cDNAs for mRNAs or miRNAs. SYBR Premix Ex Kits (TaKaRa) or miRNA Real‐time PCR Assay Kits (Aidlab Biotechnologies) were used to perform real‐time PCR accordingly. Relative amount of transcripts was calculated using the 2^−ΔΔCt^ formula. GAPDH or U6 was an internal control for mRNA or miRNA. PCR primers were listed in Table S1.

### 2.9 Caspase 3 activity assay

Caspase 3 activity was measured as described previously.^24^ Briefly, HOK cells were dissolved with a lysis buffer for 10 minutes. After centrifugation, cell lysates were collected for detection. The caspase 3 activity was tested with a fluorogenic caspase substrate Ac‐DEVD‐AFC (BioVision) and monitored at Ex.360/Em.530 using a plate reader system.

### 2.10 Terminal deoxynucleotidyl transferase dUTP nick end labelling (TUNEL) staining

Human oral mucosal tissues were fixed in 10% formalin, embedded with paraffin and cut into 4 µm sections as previously described.^24^ TUNEL staining of sections was performed by using an In Situ Detection Kit (Roche Life Science).

### 2.11 Dual‐luciferase reporter

Oral keratinocytes were culture in 24‐well plates. At 70% confluence, pGL3‐promoter plasmids (500 ng), pRL‐TK renilla luciferase reporter vector (500 ng) and IKKβ plasmids (500 ng) or miR‐122 mimic (200 nmol/L) were cotransfected into cells. After 36‐hour transfection, the luciferase activities were measured with Dual‐Luciferase Reporter Assay System from Promega. Renilla activity was served as an internal control.

### 2.12 Clustered regularly interspaced short palindromic repeats (CRISPR)/Cas9‐regulated knockout of human *VDR*

sgRNA sequence (5′‐ACGTTCCGGTCAAAGTCTCC‐3′) targeting the human *VDR* gene was cloned into lentiCRISPRv2 vector (Addgene) by using the BsmBI restriction enzyme. Packaging plasmids (pMD2.G and psPAX2) and lenti‐vector were cotransfected into HEK293T cells. Lentivirus in the culture medium of HEK293T cells was collected following 48‐hour transfection. Lentivirus particles and polybrenes (4 µg/mL) were added together into the culture medium of HOKs. Transduced cells were selected by puromycin (0.5 µg/mL).

### 2.13 Statistical analysis

Data values were presented as means ± SD. Unpaired two‐tailed Student's *t* test was selected for comparisons of two groups, and one‐way analysis of variance (ANOVA) was chosen for comparisons of three or more groups. *P* < .05 was considered to be statistically significant.

## 3 RESULTS

**FIGURE 1:**
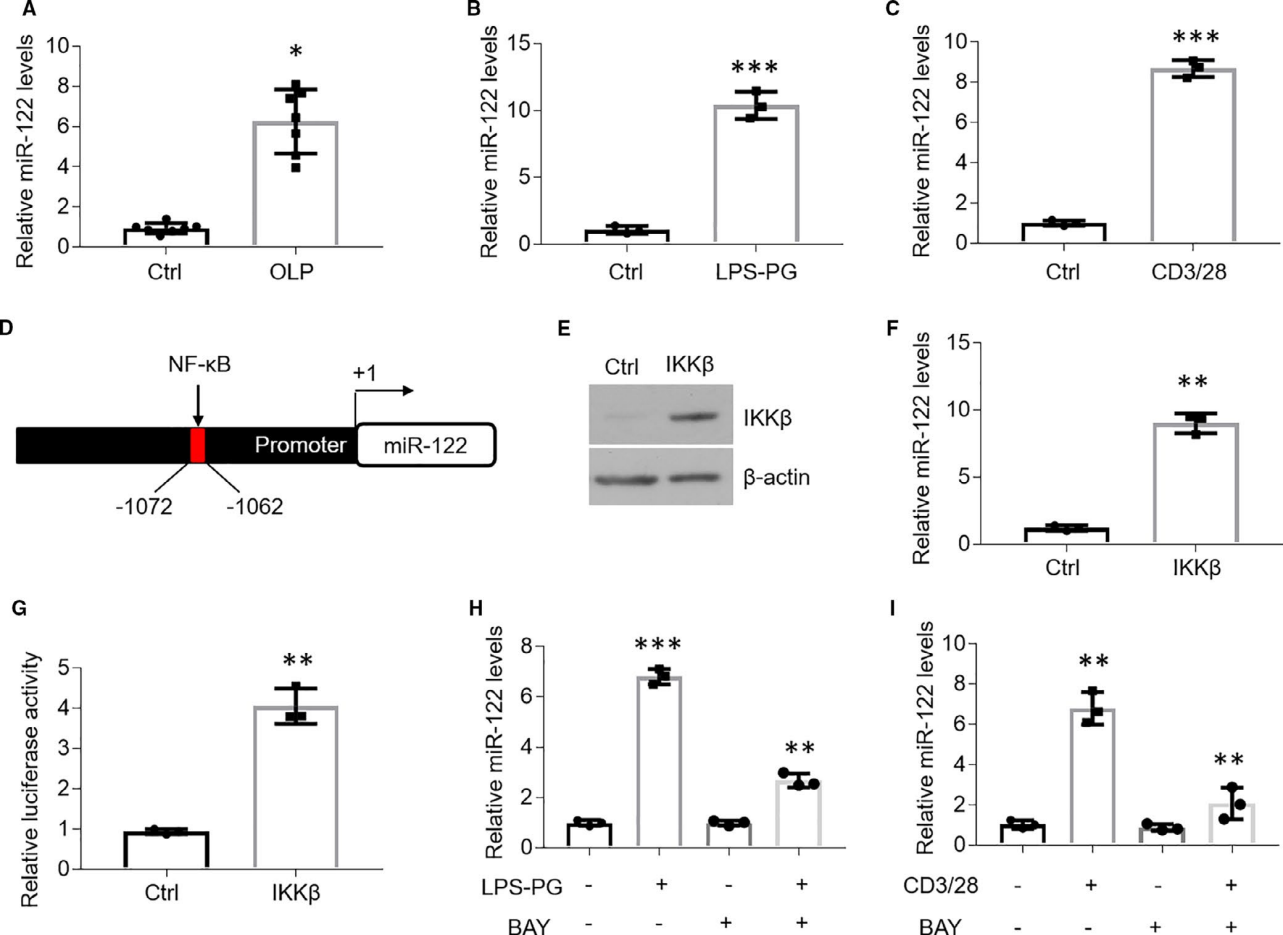
NF‐κB pathway induces miR‐122 up‐regulation in OLP. A, Real‐time PCR quantification of miR‐122 in the oral keratinocytes of human tissues, n = 7. B and C, Real‐time PCR assays showing miR‐122 expression in HOKs in the presence of LPS‐PG (B) or activated CD4+ T cells (C) treatment, n = 3. D, Promoter of human *miR‐122* gene harbouring κB element located between −1062 and −1072. E and F, Western blot (E) or real‐time PCR (F) detection of HOKs transfected with IKKβ plasmids, n = 3. G, Luciferase report assay of HOKs cotransfected with IKKβ plasmids and pGL‐3 promoter vector containing the κB element, n = 3. H and I, Real‐time PCR quantification of HOKs treated with LPS‐PG (H) or activated CD4+ T cells (I) in the presence or absence of BAY 11‐7082, n = 3. *P < .05, **P < .01, ***P < .001 vs corresponding controls. Ctrl, control; BAY, BAY 11‐7082; LPS‐PG, Lipopolysaccharide from Porphyromonas gingivalis

Levels of miR‐122 are enhanced by nuclear factor‐κB (NF‐κB) pathway in the oral keratinocytes of OLP. As miR‐122 is documented to be induced by inflammation in HCC‐derived human cell lines,^33^ we isolated the oral epithelial cells from both healthy and OLP individuals to dissect whether miR‐122 expression is increased. As shown in Figure 1, miR‐122 levels are largely increased in the oral epitheliums of OLP compared to healthy controls (Figure 1A). We next established two cell models in HOKs with LPS or activated CD4+ T cells supernatant treatment to mimic OLP in vitro as described before.^24^ In line with the results from human biopsies, we found the elevated miR‐122 expression in the two cell models upon treatments (Figure 1B,C). To explore the mechanism of miR‐122 up‐regulation, we screened the promoter region of *miR‐122* gene and identified a putative NF‐κB binding site bioinformatically (Figure 1D). After IKKβ overexpression, the status of miR‐122 is also up‐regulated in HOKs (Figure 1E,F). We then constructed a pGL‐3 promoter plasmid carrying human *miR‐122* promoter region from −2122 to −1012 bp relative to the transcription start site (TSS) and performed luciferase activity assay. As expected, luciferase activity was considerably improved in HOKs transfected with IKKβ plasmids (Figure 1G). The NF‐κB pathway inhibitor, BAY 11‐7082, reversed LPS‐ or activated CD4+ T cell–induced miR‐122 up‐regulation in HOKs (Figure 1H,I), indicating miR‐122 is mediated by NF‐κB pathway.

**FIGURE 2:**
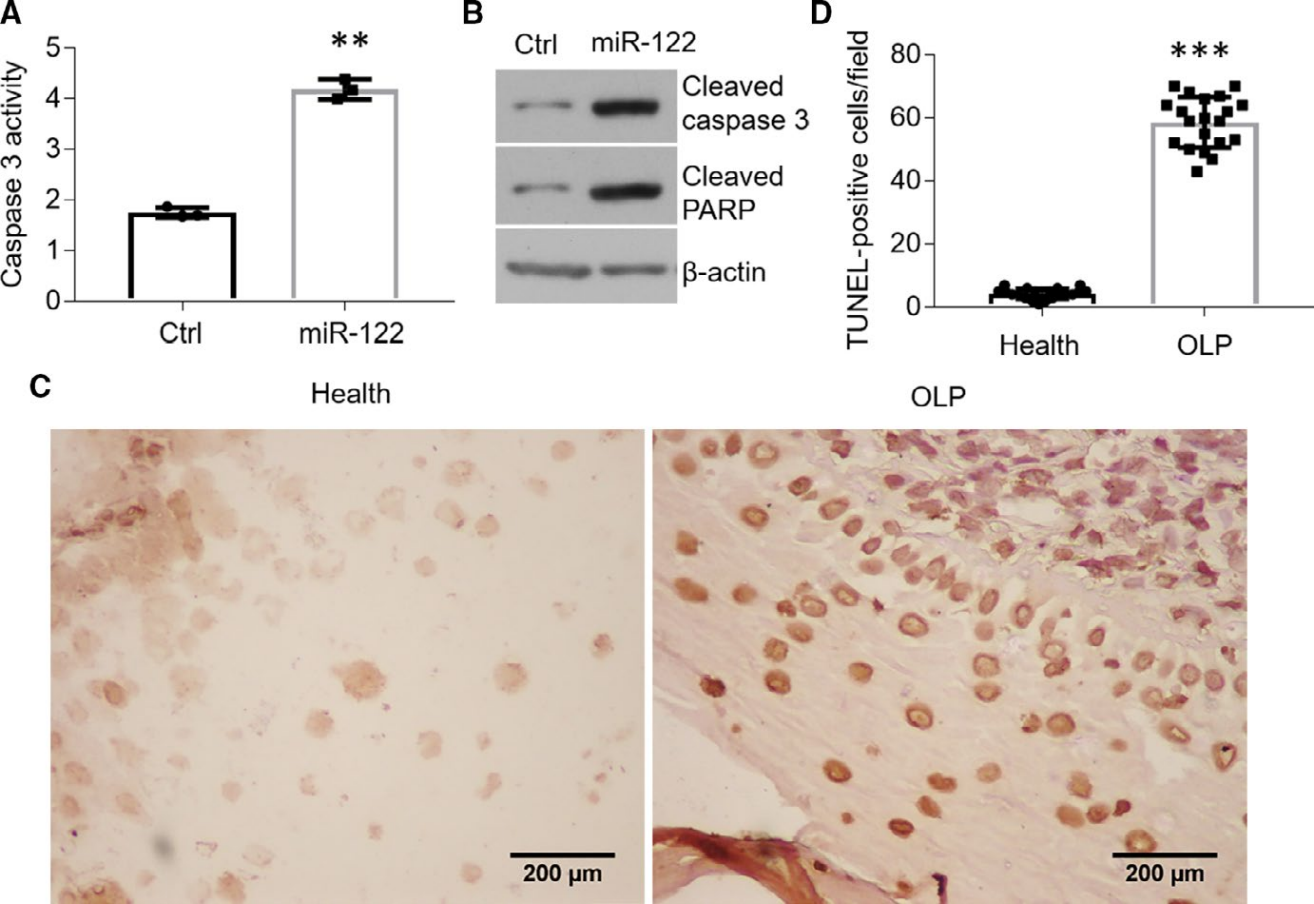
MiR‐122 triggers cell apoptosis in oral keratinocytes. A and B, Caspase 3 activity assay (A) and Western blot detection (B) of HOKs treated with miR‐122 mimics, n = 3. C and D, TUNEL staining of human oral biopsies (C) and quantification analysis (D), n = 20. **P < .01, ***P < .001 vs corresponding controls

Up‐regulation of miR‐122 induces cell apoptosis in oral keratinocytes. MiR‐122 is able to regulate cell apoptosis in a variety of cell types.^26^, ^27^ To better understand the role of miR‐122 in apoptosis of oral keratinocytes, we treated HOKs with miR‐122 mimics. As shown in Figure 2, caspase 3 activity showed a robust increase in the presence of miR‐122 mimic in HOKs (Figure 2A). Meanwhile, Western blot data exhibited that both cleaved caspase 3 and cleaved poly(ADP‐ribose) polymerase (PARP) levels were substantially increased after miR‐122 mimic treatment (Figure 2B), suggesting miR‐122 has the ability to induce cell apoptosis in HOKs. Accompanied with the up‐regulation of miR‐122 in the oral epitheliums of OLP biopsies as mentioned above, excess cell apoptosis was observed in the epithelial layers of OLP tissues compared with healthy control (Figure 2C,D). These observations suggest that miR‐122 increase contributes to cell apoptosis in oral keratinocytes.

**FIGURE 3:**
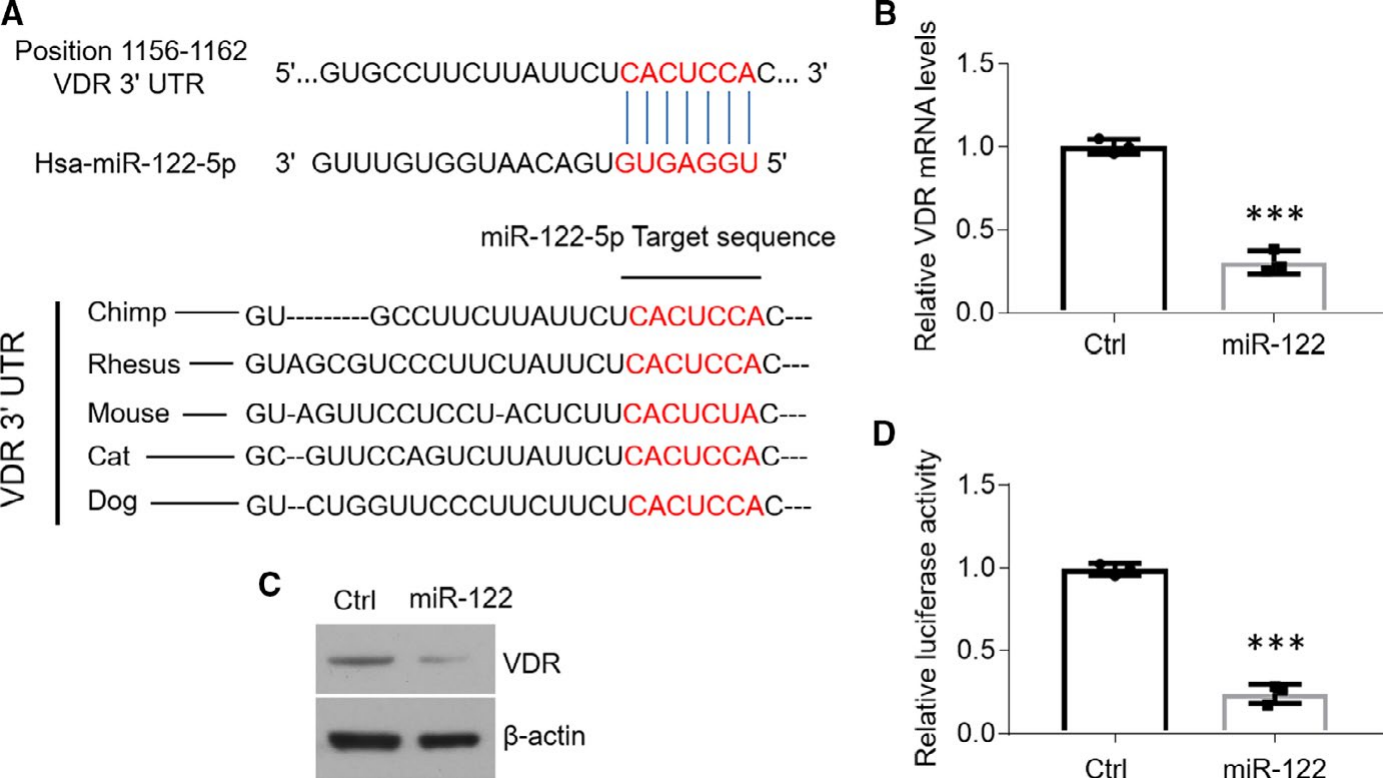
MiR‐122 targets the 3′UTR of VDR mRNA. A, The putative miR‐122 binding site in the 3′UTR of VDR mRNAs. B and C, Real‐time PCR (B) and Western blot (C) measurements of HOKs treated with miR‐122 mimics. D, Caspase 3 activity detection of HOKs treated with miR‐122 mimics. n = 3, ***P < .001 vs corresponding controls. Ctrl, control

VDR mRNA is targeted by miR‐122 in HOKs. Previous studies have demonstrated that VDR exerts a prominent function in suppressing cell apoptosis in the epitheliums of OLP.^24^ Therefore, we speculated that miR‐122 might trigger cell apoptosis through targeting VDR mRNA. Interestingly, there is a putative binding site of human miR‐122 in the 3′UTR of VDR mRNA (Figure 3A). Further analysis uncovered that the miR‐122 binding site was highly conserved across species (Figure 3A). What is more, VDR mRNA and protein levels were both decreased in the presence of miR‐122 overexpression (Figure 3B,C). To confirm it further by luciferase reporter experiment, we generated a pGL‐3 promoter plasmid carrying miR‐122 target sequence in 3′UTR fragment of hVDR cDNA (hereafter referred to as pGL3‐VDR‐3′UTR). As shown in Figure 3, miR‐122 treatment down‐regulated the luciferase activity of HOKs transfected with pGL3‐VDR‐3′UTR plasmids (Figure 3D). These data suggest that miR‐122 decreases VDR expression by target its mRNAs.

**FIGURE 4:**
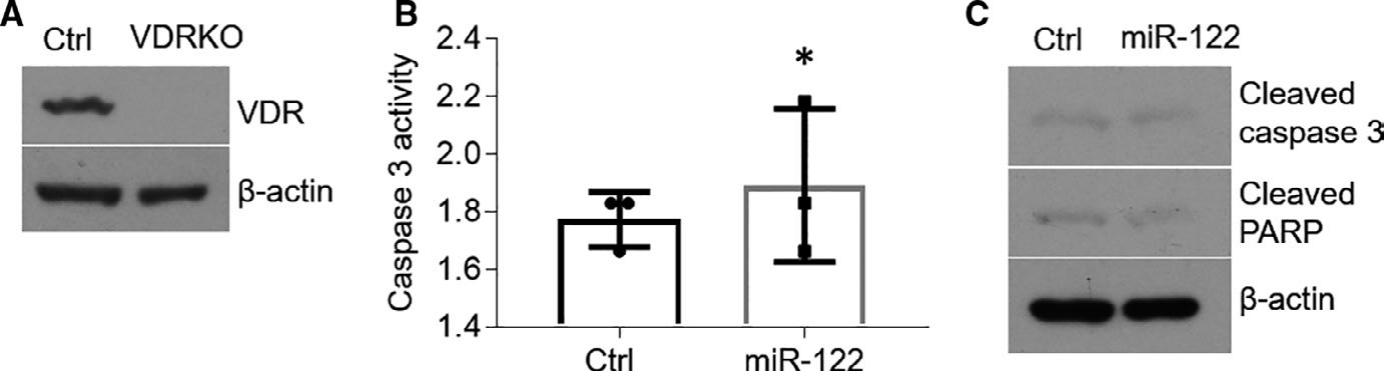
MiR‐122 induces cell apoptosis dependent on VDR. A, *VDR* in HOKs was deleted by CRISPR/Cas9 system and detected by Western blot. B and C, Caspase 3 activity (B) and Western blot (C) detections of HOKs treated with miR‐122 mimics. n = 3, *P < .05 vs corresponding controls. Ctrl, control

The regulation of miR‐122 on cell apoptosis is dependent on VDR in HOKs. To explain whether miR‐122 regulates oral keratinocytes apoptosis dependent on VDR, we deleted the *VDR* gene in HOKs using CRISPR/Cas9 system. Western blot data displayed that VDR expression was eliminated in human cell line (Figure 4A). We then detected cell apoptosis‐related factors in VDR knockout (VDRKO) cell line. As shown in Figure 4, miR‐122 overexpression failed to elevate caspase 3 activity and expression of cleaved caspase 3 and PARP in VDRKO HOKs (Figure 4B,C). These data suggest that the function of miR‐122 in cell apoptosis relied on the existence of VDR.

**FIGURE 5:**
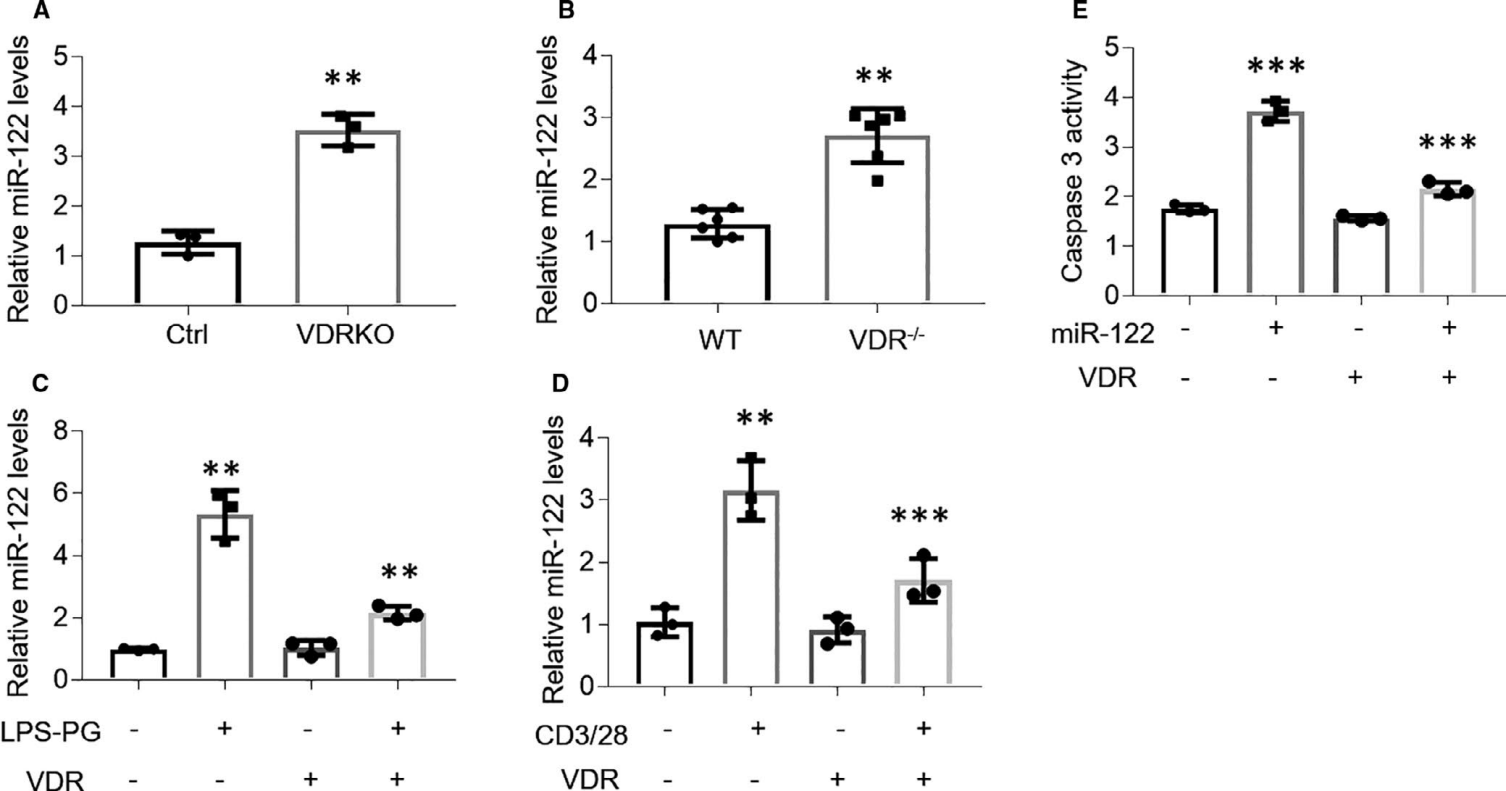
VDR is able to suppress miR‐122 expression in HOKs. A and B, Real‐time PCR tests of miR‐122 expression in VDRKO HOKs (A) or VDRKO mouse oral keratinocytes (B). C and D, VDR plasmids‐transfected HOKs were treated with LPS‐PG (C) or activated CD4+ T cells (D) and miR‐122 expression was tested by real‐time PCR. E, Caspase 3 activity detection of HOKs treated with miR‐122 mimics in the presence or absence of VDR plasmids transfection. n = 3. **P < .01, ***P < .001 vs corresponding controls. Ctrl, control; VDRKO, VDR knockout; LPS‐PG, Lipopolysaccharide from Porphyromonas gingivalis

VDR has a role in inhibiting miR‐122 up‐regulation in HOKs. Since VDR protein is capable of binding with IKKβ physically to suppress active NF‐κB pathway,^34^ we reasoned that VDR might control miR‐122 expression via regulating NF‐κB pathway. To this end, we detected miR‐122 levels in both human and mouse VDRKO oral keratinocytes. As shown in Figure 5, miR‐122 expression was enhanced in human and mouse VDRKO oral keratinocytes (Figure 5A,B). Moreover, VDR overexpression blocked miR‐122 increases in HOKs upon LPS or activated CD4+ T cells challenge (Figure 5C,D). Accordantly, miR‐122–induced up‐regulation of caspase 3 activity was also ameliorated in HOKs transfected with VDR plasmids (Figure 5E).

## 4 DISCUSSION

Oral lichen planus, an inflammatory disease dominated by T cells, shows oral discomfort which impairs the life quality of patients.^3^ Due to the lack of detailed cell machinery for OLP pathogenesis, there are still no effective strategies in clinic for management.^3^ For symptomatic patients, management includes oral hygiene maintaining, jagged dental surfaces elimination and irritating foods avoidance.^35^ For controlling substantial and lingering symptoms, corticosteroids are usually applied topically.^35^ The present study investigated the roles of miR‐122 in OLP and showed that miR‐122 induces cell apoptosis in oral keratinocytes via targeting VDR mRNA. The massive cytokines or chemokines produced by lymphocytes in the lamina propria of OLP may activate NF‐κB pathway in oral keratinocytes, leading to miR‐122 overexpression and VDR reduction in the epithelial layer. Our work provides novel insights into the molecular underpinning whereby miR‐122 regulates the development of OLP.

MiR‐122 is secreted into the blood stream as a predictive indicator for chemical‐, alcohol‐ and viral‐induced liver injury,^36^, ^37^ but its functions in oral diseases are poorly understood. Here, we found miR‐122 expression is increased in the oral keratinocytes of OLP by real‐time PCR, consistent with other studies reporting miR‐122 is induced by inflammation.^33^ In agreement with the data from biopsies, miR‐122 expression in HOKs challenged with LPS or activated CD4+ T cells is also up‐regulated robustly. Since antagomiR‐122 injection can relieve inflammation‐induced anaemia in mice,^33^ strategies to block miR‐122 might preclude the onset or development of OLP in patients. However, some studies suggest that miR‑122 levels are considerably reduced in the peripheral blood mononuclear cells derived from OLP patients.^38^ This discrepancy might result from the different OLP subtypes or progression involved in these investigations.

Aberrant apoptosis, a typical characteristic in the epithelial layer of OLP, impairs the integrity of oral epithelia barrier against bacteria.^28^, ^29^ In this study, we verified that miR‐122 overexpression leads to excess cell apoptosis in HOKs, which is in accordant with previous observations indicate that miR‐122 regulates apoptosis in various cell types and diseases.^26^, ^27^ In addition, consistent with early reports,^24^ substantial TUNEL‐stained oral keratinocytes were observed in the specimens from OLP patients. Therefore, targeting miR‐122 in the context of OLP might be a brand‐new approach to balance cell apoptosis in the oral epitheliums of this disorder.

Recent work has predicted that VDR mRNA is a potential target of has‐miR‐122.^39^ Because we claimed that VDR plays a protective role in regulating cell apoptosis in oral keratinocytes,^24^ it could be very interesting to explore the relationship between VDR and miR‐122 in the setting of OLP. In line with previous predicted work,^39^ we confirmed miR‐122 treatment decreases VDR mRNA levels by real‐time PCR and luciferase report assay in HOKs. We also confirmed that miR‐122 triggers cell apoptosis in oral keratinocytes dependent on VDR, which is different with other studies suggesting miR‐122 regulates cell apoptosis through targeting these pathways such as SIRT6‐elabela‐ACE2 signalling and PLD1.^26^, ^27^ In accordant with previous investigations,^33^ we provided compelling evidence that miR‐122 levels are enhanced by active NF‐κB pathway under inflammatory condition. Since VDR is demonstrated to inactivate NF‐κB pathway by interacting with IKKβ physically,^34^ VDR overexpression is potential to reduce miR‐122 inversely by suppressing NF‐κB pathway. Indeed, our data showed that VDR plasmids transfection blocks miR‐122 and caspase 3 activity increases under inflammatory condition, indicating a complex and interactive biological system in the oral keratinocytes of OLP.

**FIGURE 6:**
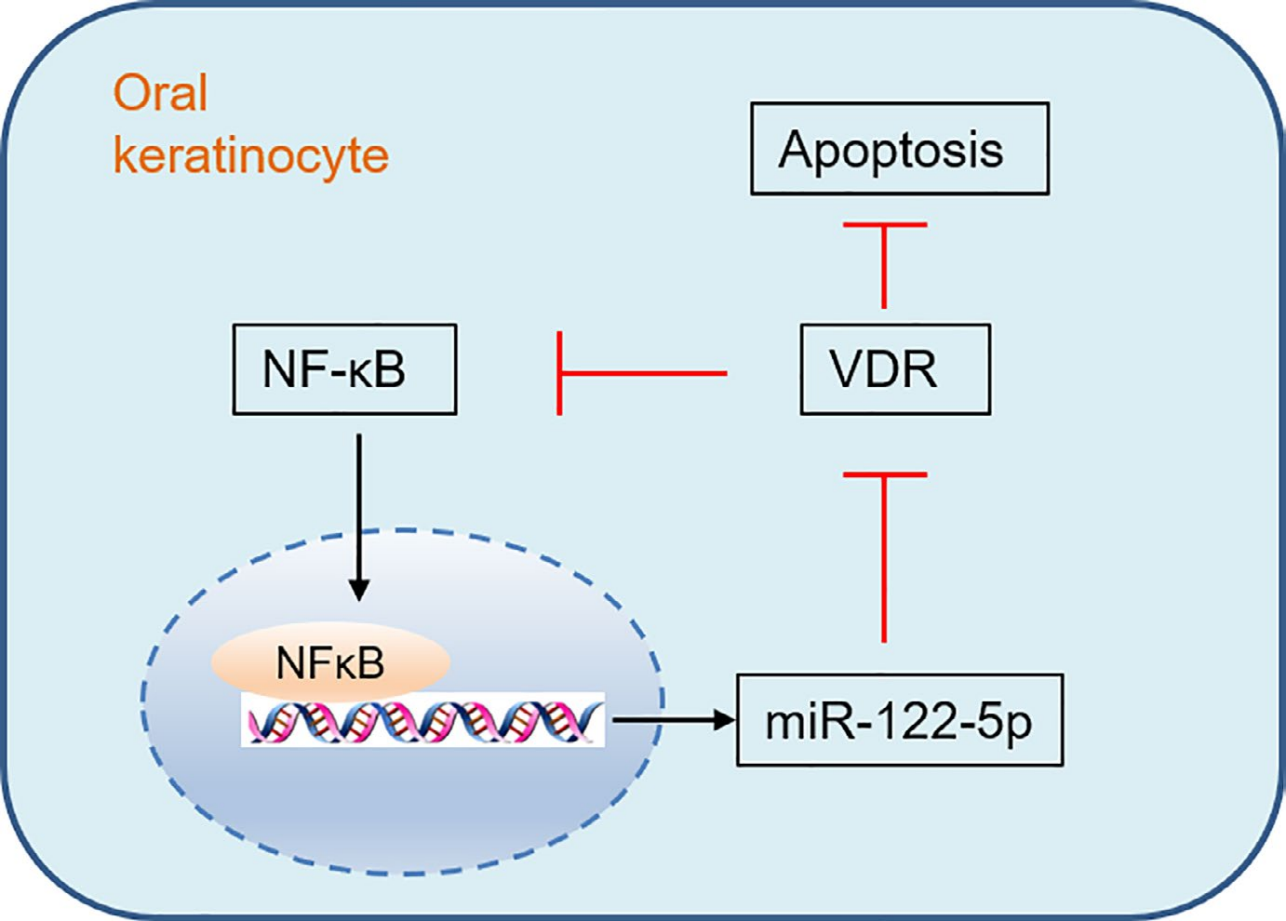
Schematic illustration of the role of miR‐122‐5p in VDR

In conclusion, we present evidence that NF‐κB pathway‐induced miR‐122 up‐regulation exacerbates cell apoptosis in oral keratinocytes by decreasing VDR expression (Figure 6). Targeting miR‐122 might be an effective strategy for OLP management. However, more experiments are warranted to prove the efficiency of this new approach.

## CONFLICT OF INTEREST

The authors confirm that there are no conflicts of interest.

## AUTHOR CONTRIBUTIONS

**Xuejun Ge:** Investigation (equal). **Hanting Xie:** Investigation (equal). **Lu Wang:** Investigation (equal). **Ran Li:** Resources (equal). **Fang Zhang:** Resources (equal). **Jing Xu:** Resources (equal). **Bin Zhao:** Project administration (equal). **Jie Du:** Conceptualization (equal); Funding acquisition (equal); Writing‐original draft (equal); Writing‐review & editing (equal).

## Supporting information

Table S1‐S2

## Data Availability

Our data are available. Please contact the corresponding author for requirement.
