# Supplementary material for: MicroRNA‐122 promotes apoptosis of keratinocytes in oral lichen planus through suppressing VDR expression
Source: J Cell Mol Med. 2021 Mar 3;25(7):3400–7. doi: 10.1111/jcmm.16418 (PMC8034474; doi:10.1111/jcmm.16418)
Supplement: Supplementary file 1 — Table S1‐S2 [file JCMM-25-3400-s001.docx]

**Supplemental tables**

Supplemental table 1. Primer sequences in this work

| Primer name | Forward (5’-3’) | Reverse (5’-3’) |
| --- | --- | --- |
| Hsa-mir-122 | TGGAGTGTGACAATGGTGTTTG |  |
| Mmu-mir-122 | TGGAGTGTGACAATGGTGTTTG |  |
| U6 | GATGACACGCAAATTCGTGAA |  |
| hVDR | GACTTTGACCGGAACGTGCCC | CATCATGCCGATGTCCACACA |
| hGADPH | ACCACAGTCCATGCCATCAC | TCCACCACCCTGTTGCTGTA |
| pGL3-promoter  -3’UTR-VDR | CGGGTGGCTAGTCAGAGAGA | AAGTTGTATCACTCCGCCCC |
| pGL3-promoter  -miR-122 | GAACGACTTTCAGCTTTCGCA | CGCCTTATTCTCATGTAGATCCTG |

Supplemental table 2. Antibodies information

| Antibodies | Source | Identifier |
| --- | --- | --- |
| VDR | Santa Cruz | Cat# sc-13133 |
| Anti-IKKβ | Cell Signaling | Cat# 8943 |
| Cleaved caspase 3 | Cell Signaling | Cat# 9654 |
| Cleaved PARP | Cell Signaling | Cat# 9541 |
| β-actin | Santa Cruz | Cat# sc-47778 |
